# Descriptive Epidemiology of New Zealand’s Highest Mortality Earthquake: Hawke’s Bay in 1931

**DOI:** 10.1038/s41598-019-41432-6

**Published:** 2019-03-20

**Authors:** Christine Clement, Shannon Abeling, Joanne Deely, Andrea Teng, George Thomson, David Johnston, Nick Wilson

**Affiliations:** 1 Genealogist/Family Historian, Te Puke, Bay of Plenty New Zealand; 2 Department of Civil and Environmental Engineering, University of Auckland, Auckland, New Zealand; 3 Canterbury District Health Board, Christchurch, New Zealand; 4 Department of Public Health, University of Otago Wellington, Wellington, New Zealand; 5 GNS Science and Massey University, Wellington, New Zealand

## Abstract

In this study we aimed to produce the first detailed analysis of the epidemiology of the severe injury and mortality impacts of the 1931 Hawke’s Bay earthquake in New Zealand (NZ). This involved the compilation and analysis of archival data (hospitalisations and deaths) including the examination of 324 death certificates. We found that there were 662 people for whom some hospitalisation data were available at four weeks post-earthquake: 54% were still in hospital, 4% were still classified as “serious”, and 5% had died (n = 28). Our classification of death certificate data indicated 256 earthquake-attributable deaths and for another five deaths the earthquake was estimated to have played an indirect role. There were 15 buildings associated with three or more deaths each (accounting for 58% of deaths with a known location). Many of these buildings were multi-storey and involved unreinforced masonry – with some of this falling into the street and killing people there (19% of deaths). In contrast, deaths in homes, which were typically of wood construction and single stories, comprised only 3% of deaths. In conclusion, this earthquake had a relatively high injury impact that appears partly related to the lack of regulations for building construction that would mitigate earthquake-related risk. Such regulations continue to be of relevance for New Zealand and for other countries in earthquake zones.

## Introduction

The Hawke’s Bay earthquake on 3 February 1931 is one of the top two sudden mass fatality events in the 1900 to 2015 period for New Zealand and is the highest mortality earthquake in the country’s recorded history^1^. It is also probably the worst *sudden natural hazard event* to strike New Zealand. The earthquake was of a high magnitude (M_W_ 7.4–7.6)^2^ and a brief description of it follows:

“Occurring at 10:47 am (NZ Summer Time) on Tuesday, 3 February 1931, the Hawke’s Bay earthquake (also known as the Napier Earthquake) is recorded as having caused the largest loss of life and most extensive damage of any quake in New Zealand’s history. The effects of the earthquake were greatest in the towns of Napier and Hastings, but other towns in the Hawke’s Bay also suffered major damage.” There was … “extensive fissuring, slumping and landslides occurring over much of Hawke’s Bay” …. “The earthquake was followed by fires in the business areas of both Napier and Hastings that became uncontrollable as water pressure dwindled because of broken water mains. The Ahuriri Lagoon in Napier was raised drastically in the earthquake, changing the coastline significantly ….”^3^.

Also of note was the severe damage to local hospitals and the need to transfer patients to other hospitals around the lower North Island of New Zealand: “Napier’s hospitals were badly damaged and unusable, so patients were moved to the lawns of the Botanical Gardens, where a surgical station was set up. Emergency hospitals were set up at the Hastings racecourse and at Napier Park racecourse, where doctors operated beneath the grandstand. In the ensuing days, many injured people were evacuated to other centres”^4^.

Previous work suggests a range in the reported death toll for this earthquake, variously reported as: 251^5^; 255^6^; 256^3^; 258^1^; 260 (range: 256 to 266)^7^; and 261 (range: 257 to 273)^8^. Some individuals with earthquake-related injuries appeared to have died of injuries in subsequent weeks and their names were not included in the official records (often after being moved in an injured state to other cities/towns). Basic descriptive epidemiology of this disaster is also limited^8,9^ eg, with no description of the socio-demographic characteristics of those who died and with no consideration of death certificate data.

Given the above, we aimed to produce the first detailed analysis of the descriptive epidemiology of the severe injury and mortality impacts of this 1931 Hawke’s Bay earthquake. It was anticipated that this work should contribute to a richer understanding of this major disaster, and potentially help to improve the prevention and management of future disasters in New Zealand and other countries that still lack building regulations and are in earthquake zones.

## Methods

### Morbidity data

We obtained archival reports on patient status for all patients “admitted from earthquake area to departmental institutions” that were compiled four weeks after the earthquake^10,11^. This included public hospital data from beyond Hawkes Bay, but did not cover those in private hospitals. Missing data (eg, on age in 1931) were obtained from other sources (eg, birth records, death records, and other genealogical/history databases: ancestry.com.au, bdmhistoricalrecords.dia.govt.nz, paperspast.natlib.govt.nz and familysearch.org). Some extra detail on people hospitalised with injuries came from police and relief fund archival files^12,13^ along with contemporary newspaper articles.

### Mortality data

We obtained 324 electronic versions of death certificates, mainly from purchasing these from “Birth Deaths and Marriages” (Department of Internal Affairs). These were for all names associated with the potential earthquake deaths as listed in various sources: contemporary newspapers, books on the earthquake with lists of names, the names on monuments in Napier and Hastings, and based on: the age at death, time, burial date and location, and family history information. We also identified deaths among hospitalised cases^10^ and obtained death certificates for these cases. Additional information on the circumstances of some of the deaths (eg, the location of death and the role of fire) came from “Affidavits to Death” contained in Probate records held on a genealogical database site (https://www.familysearch.org). Published newspaper obituaries (identified in *Papers Past*)^14^ also provided additional information on selected cases.

From the above data sources we were typically able to code the location of injury or death, to inside specific buildings or on the street outside specific buildings. Failing that, we used death certificate data which sometimes included site details, although this was also sometimes at non-specific levels of just a street name or at a whole suburb/town level.

### Assignment of death as being earthquake-related

We classified deaths as being directly earthquake-related if the death certificate stated this or if the cause of death on the death certificate and other available information was suggestive that an earthquake-related cause was dominant “on the balance of probabilities” (for details see Supplementary Material, Tables S1 to S3). In addition, we included sudden cardiovascular deaths when there was evidence that these were related to earthquake-induced stress. In considering the likely role of the earthquake, we took into account the evidence of earthquakes increasing the risk of sudden cardiac death on the day of an earthquake^15^ and in subsequent days and weeks (takotsubo cardiomyopathy)^16^. Also included were deaths for people reported as “officially missing” on death certificates and who were thought to have been in the earthquake area. This included names not definitively linked to an unidentified body or charred human remains.

We classified other deaths as *indirectly* earthquake-related if these included the following (with details in Supplementary Material, Table S2):

- Deaths amongst those people with likely pre-existing conditions in the days, weeks and months after the earthquake where these people did not have serious injuries or “shock” reported on the death certificate.
- Deaths amongst people involved in aid and rescue efforts but which were unrelated to building collapses (eg, from a plane crash at Wairoa that killed three people).

Classification was performed by the two medical practitioners (NW and AT) in the research team and any disagreements in classification was resolved by further discussion. The process of refining our classification approach was iterative and required some discussion to achieve consensus on the final classification of cases, thus we did not attempt any formal assessment of inter-rater reliability.

### Analysis

Results were analysed in EpiInfo and OpenEpi. We calculated severe injury and mortality rates per 1000 population for the combined urban area of Napier for Hastings and also the geographical area of Hawke’s Bay (as per the population estimates for April 1931 and including the Māori population)^17^. Given an absence of age-group/sex estimates for these areas, the following steps were performed:

- The age-distributions from the census results for 1926 and 1935 (since no census was held in 1931)^18^, were used to interpolate the age-structure of the national population for 1931 (albeit European only, given the lack of Māori data in this Yearbook-based data).
- These national data were then used to estimate the age and sex structure in 1931 for the urban population of Napier and Hastings.

### Ethical approval

University of Otago Human Ethics Committee approval (reference number D18/342). We note that all the historical mortality data (including death certificates) are already publicly available. Nevertheless, we have only presented anonymised data in our results.

## Results

### Injuries involving hospitalisation

The hospitalisation data reported by the Department of Health are shown in Tables 1 and 2, and Supplementary Material, Table S4. Patients were dispersed to hospitals in 14 towns and cities outside of Napier and Hastings, with almost half taken to Palmerston North and Wellington (Table S5). These data are of constrained utility because undefined numbers of patients did not have earthquake-related injuries, but rather were pre-earthquake hospital in-patients who subsequently needed to be transferred due to the severe damage to the Hawke’s Bay hospitals. Nevertheless, at four weeks after the earthquake, most (54% of the original total) were still in hospital and 23 (4%) were still classified as “serious” (Table 1). A total of 28 had died in hospital (5%).

**Table 1:** Status of hospitalised cases reported by the Department of Health as of 4 March 1931 (4 weeks after the earthquake, and for whom the status was known in the relevant hospitals [see Table S5]).

Description of status of cases*	Number	Percentage
“Discharged”	257	41.9
“Died”	28	4.6
***Still in hospital***		
“Satisfactory”	266	43.4
“Serious”	23	3.8
“Other” (eg, “improving”)	39	6.4
**Total**	**613**	**100**

*These records do not indicate how such terms as “satisfactory” and “serious” were defined by the hospital staff.

**Table 2:** Type of earthquake-related injuries for 377 hospitalised cases for which there was specific injury data.

Type of injury	Number of patients	Percentage	Deaths reported by 4 March 1931
Fracture/s** (n = 73); fracture/s** + other* (n = 19) (for details see Table S6)	92	24.4	1
“Shock” (n = 62)***; “shock” + other* (n = 13)	75	19.9	5
Head injury (n = 24); head injury + other* (n = 13)	37	9.8	2
Contusion (including “abrasion” or “bruising”)	30	8.0	1
Laceration (n = 24); laceration + other* (n = 6)	30	8.0	0
Lower limb injury (no fracture)	27	7.2	0
Other injury (eg, electrocution)	19	5.0	0
Abdominal injury (n = 5); abdominal injury + other* (n = 5)	10	2.7	1
Upper limb injury (no fracture)	10	2.7	1
Back injury (n = 5); back injury + other* (n = 4)	9	2.4	0
Paralysis/spinal injury; spinal injury + other* (n = 1)	9	2.4	0
Amputation (likely for limb fractures or crush injuries)	6	1.6	0
Burn (n = 4); burn + other* (n = 1)	5	1.3	0
Cerebral haemorrhage (n = 4); cerebral haemorrhage + other* (n = 1)	5	1.3	4
Crush injury	5	1.3	0
Chest injury (n = 3); chest injury + other* (n = 1)	4	1.1	0
Injured pelvis (n = 2); injured pelvis + other* (n = 2)	4	1.1	0
**Total**	**377**	**100**	**15** ^**#**^

*“Other” typically refers to a less serious additional injury (eg, contusion).

**Fracture/s (included rib fractures; but in this table it excludes skull fractures and paralysis/spinal injuries: with these included in other categories in this table, ie, the “head injury” and “paralysis/spinal injury” categories respectively). This category also includes multiple fractures eg, to an arm and to a leg. (See Table S6 for more details on fractures).

***It is unclear how this term was being used by those assigning this diagnosis eg, psychological shock or also including haemorrhagic shock and even cardiogenic shock.

^#^Of these 15 – based on death certificate data (and other information eg, see Table 3), we classified only 8 as primarily being earthquake-related deaths.

The most common type of injury was fracture, followed by “shock”, and head injury (Table 2). But the term “shock” did not differentiate between possible forms of shock (eg, psychological or haemorrhagic). More males than females suffered “shock” (rate ratio = 1.12, 95%CI = 1.01 to 1.25; p = 0.0335, two-tailed test), but the age distribution for this injury was similar to that of other injuries. There were also cases of sudden death from cardiac events in people without physical injuries (eg, see details for “person A” in Supplementary Material, Table S1).

Lower limb fractures were twice as common as upper limb ones (Table S6) and 13% of fractures were compound ones. Despite widespread post-earthquake fires, only a small number of hospital patients had burn injuries (Table 2).

**Figure 1:**
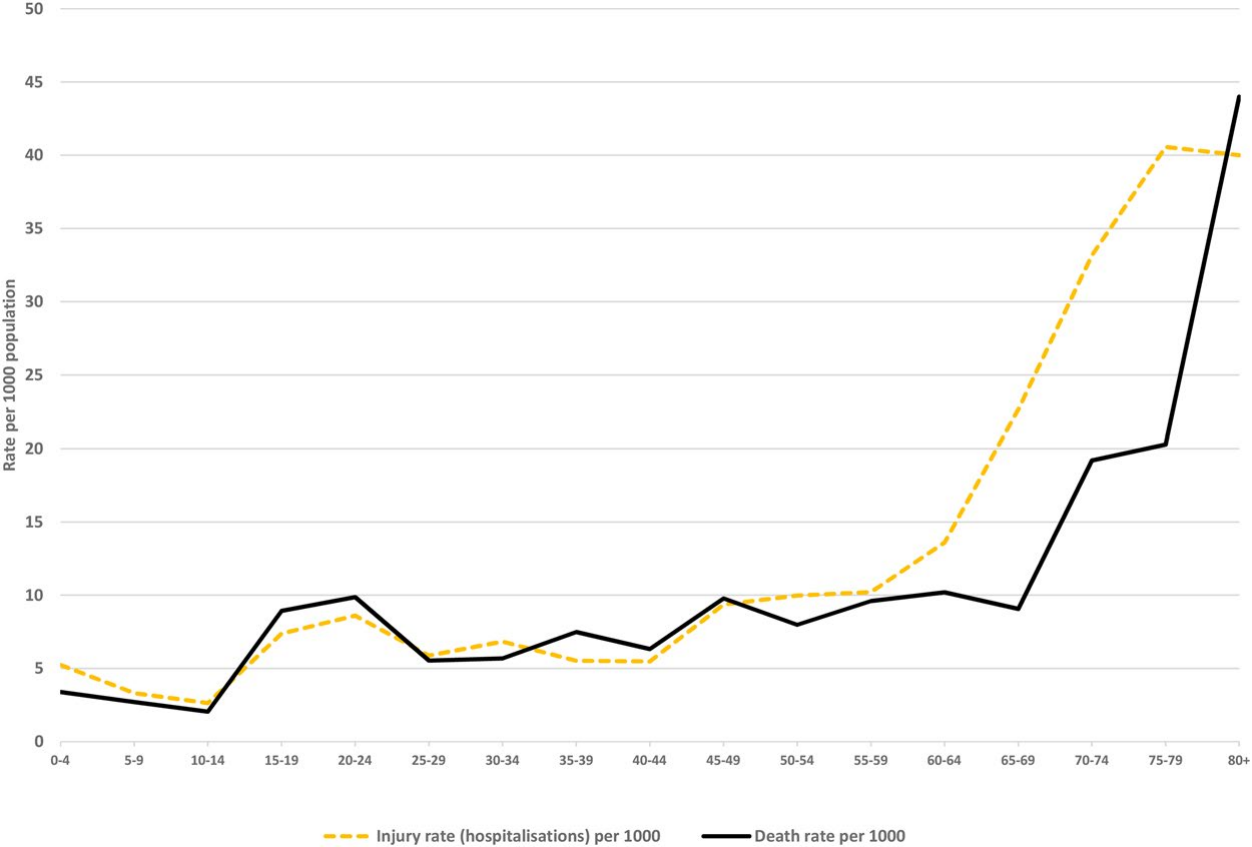
Age distribution of earthquake-related hospitalisation and deaths (per 1000 population).

The median age of the injured people was 42.5 years (Table S7) with the most commonly injured age-group being 20–24 years (Table S8). The 0–14 year age-group comprised 13% of cases and the 65 + age-group comprised 22% of cases. The injury rate was significantly higher in the 60+ year age-group (Fig. 1, Table S8). It was also 17% higher for males than females, but this was not statistically significant (Table S7).

The assumed geographical location of where the person was injured was dominated by Napier (69% for the modern-day Napier City Council area), followed by Hastings (22% for the Hastings District Council area) (Table S9).

There was one person hospitalised as a result of being injured during rescue work. A police report also indicated that some police officers were “slightly injured” in the rescue efforts^19^.

### Earthquake-related mortality

The analysis of the death certificate data suggested a total of 256 deaths occurred during earthquake shaking (Table 3, S1–S3). This total included 17 names that were not on previous lists of earthquake victims, while we also discounted 14 names that were on such lists (see footnotes to Table 3 for these lists). Furthermore, we estimate an additional five indirect deaths in which the earthquake probably contributed to the risk of death but was probably not the predominant cause of death (Table 3). In another three cases the information was too unclear to make an assessment. To facilitate comparisons with other earthquakes we estimated both the injury rate (for hospitalised injuries) and the mortality rate (Table 4).

**Table 3:** Classification of earthquake-related deaths based on death certificate wording, including relationship to previously classified deaths.

Description	Number	Percentage
“**Killed** during earthquake – Coroner considered inquest unnecessary”	128	50.0
“The verdict of the coroner’s jury was that death occurred through **collapse of building** caused by earthquake of great severity”	89	34.8
**Injury sustained** during earthquake with the Coroner mentioning “earthquake” in the certificate eg, “Coroners Verdict – Died from a fractured skull as the result of being struck on the head by a tank overthrown by an earthquake”	6	2.3
Reference made to the earthquake and an **injury** (eg, a fracture) or to “shock” (but no reference to the Coroner)	11	4.3
Death certificate did not refer to the earthquake or the Coroner but did refer to either a **fracture or paraplegia**. Some of these cases are discussed in Supplementary Material, Table S1.	6	2.3
Reference to a “**cerebral haemorrhage**” in the post-earthquake period, but with no specific reference to the earthquake or head injuries. See also Supplementary Material, Table S1.	4	1.6
Reference to a post-earthquake **cardiovascular event** (n = 5) or other potentially earthquake-related cause (n = 1). See also Supplementary Material, Table S1.	6	2.3
Reported as: “**Officially missing**”. For all these cases there appears to be a strong assumption by the authorities that the person died in the earthquake damaged area ie, with some of these people being associated in some inconclusive way to the 4 unidentified bodies recovered or to six collections of charred human remains or body parts (eg, feet) that were never conclusively identified.	5	2.0
**Unidentified bodies** which did not match any descriptions of the known missing people (ie, a woman aged around 70 years)	1	0.4
**Total**	**256**	**100.0**
***Previously recognised as earthquake related***		
On a **former list** of earthquake victims* (ie, all but 17)	239	93.4
Listed on either the Napier or Hastings earthquake **memorials**	192	75.0
***Indirect deaths***		
**Indirect deaths** where the earthquake probably contributed but which was not considered the major cause of death (see Supplementary Material, Table S2).	2	—
**Aircraft crash** in Wairoa as part of aid efforts after the earthquake	3	—
***Too unclear to determine an earthquake role***		
**Inadequate information** to make an assessment (see Supplementary Material, Table S3).	3	—
Listed on previous lists* of earthquake victims – but discounted in our analysis		
Cases where the death certificate and other information indicated that the death was **not likely to be earthquake related**. (However, in one case we re-classified to “indirect” (Table S2) and in two cases to “unclear” (Table S3) – as per the directly above row in this table.	14	—

*As per the “Earthquake victims list” held by MTG Hawke’s Bay in Napier (museum, theatre and gallery), the “Hastings Casualty List” held by the Hastings Library, and the book “The Shock of ‘31”^37^.

**Table 4:** Severe injury and mortality rates in the 1931 Hawke’s Bay earthquake.

Key rate/proportion	Using the Napier and Hastings urban area population (1931) as the denominator (n = 36,050)	Using the Hawke’s Bay geographical area population (1931) as the denominator population (n = 45,895)
Injury rate for injuries involving hospitalisation (per 1000 population)	10.5 (377/36,050; as per Table 2)*	8.2 (377/45,895; as per Table 2)
Death rate (per 1000 population)	7.1 (256/36,050; as per Table 3)	5.6 (256/45,895; as per Table 3)
Proportion of hospitalised cases dying	2.1% (8/377) (see Table 2)	

*This may be an underestimate given the larger number of potentially hospitalised injured detailed in Table S4 (but with a proportion of these being from non-earthquake related conditions).

The deaths occurred predominantly on the day of the earthquake (87%) and then in the subsequent week (6%) (Table 5). There were four deaths occurring after the one-year anniversary of the earthquake (eg, sequelae associated with paraplegia, see Table S1).

**Table 5:** Key characteristics of the earthquake-attributable deaths.

Characteristic	Number	Percentage
***Timing of death (from death certificates)***		
Day of the earthquake (3 February 1931)	223	87.1
Within subsequent week (to 10 February 1931)	14	5.5
Weeks 2 to 4 (11/2/31 to 3/3/31)	4	1.6
In subsequent year (after week 4)	11	4.3
More than a year later	4	1.6
**Total**	**256**	**100.0**
***Person-related***		
Male (from death certificate)	134	52.3
Female (from death certificate)	122	47.7
Mean age in years (range) – based on death certificate or various genealogical sources (there was no statistically significant differences between males and females, 40.8 vs 37.3 years respectively)	38.9 (1 day to 92 years)	—
Median age in years (for age-distribution see Figs 1 and 2, and Table S8)	37	—
Suggestive evidence that the person was buried at a marae (so probably of Māori ethnicity)	2	0.7
***Person-related, birth location (missing for n = 8) (genealogical sources)***		
Born in Hawke’s Bay	91	36.7
Born elsewhere in NZ	70	28.2
Born overseas	87	35.1
**Total**	**248**	**100**

**Figure 2:**
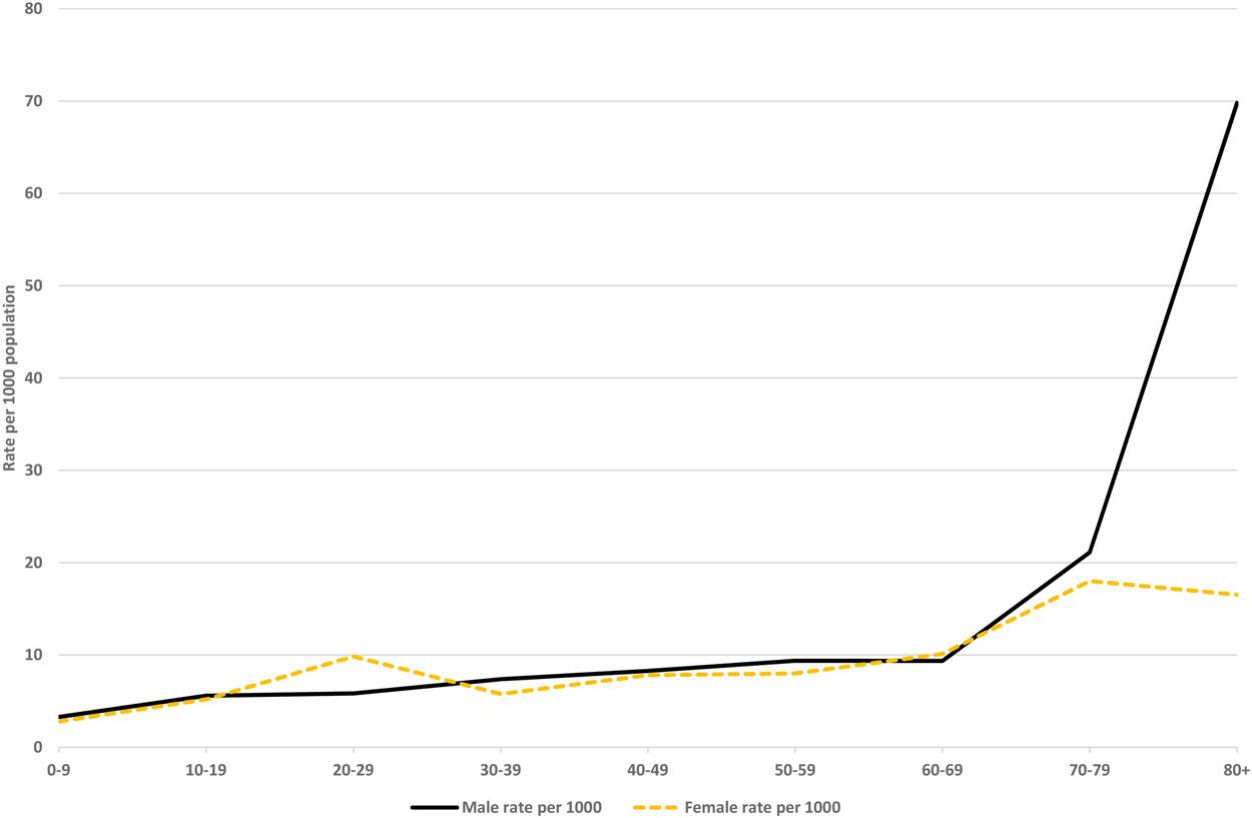
Age and sex distribution of earthquake-related deaths (per 1000 population).

The median age of death was 37 years but with the range being from one day old to 92 years. For most age-groups, the mortality rate was fairly similar to the hospitalised injury rate, except the latter was generally higher in the 60+ age-group (Fig. 1). The mortality rates were also fairly similar for males and females in each age-group (Fig. 2), except it was higher in the 80+ year age-group in men. The latter was the only statistically significant difference in the male vs female mortality rates (at p = 0.0447).

Table 6 considers different demographic and occupational groupings and their likely location at the time of the earthquake. There were relatively high numbers of deaths among workers who were likely to have been in their workplaces: ie, in retail/shop workers at 20% of all deaths and office workers at 9% of all deaths. The relatively high proportion of deaths occurring amongst those in hospital (9% of all deaths) and in a nurses home (4%) also stood out. In contrast there were very low number of deaths in those with a rural occupation at under 1% of deaths.

**Table 6:** Demographic and occupational groupings for earthquake-related deaths of relevance to likely locations at the time of the earthquake.

Characteristic (n = 1 not classifiable)	Number	Percentage
***Possibly was at home at the time of the earthquake (but some may have been elsewhere eg, at a commercial/retail district at the time of the earthquake)***		
Aged under 5 years (and outside of hospital or nursing homes)	9	3.5
Aged 65+ years*	17	6.7
“Married”/“spinster”/“widow” (if not 65+ years)**	46	18.0
Probably at an educational institution at the time of the earthquake***		
Aged 5–14 (at ages 12–14 years many would have left school and we excluded those with a stated occupation eg, “drapers assistant”)	15	5.9
Aged 15+ and described as “student”	12	4.7
Probably at a workplace at the time of the earthquake (but some may have been on leave etc)**		
Retail/shop worker (eg, shop assistant [n = 9], draper [n = 5], draper’s assistant [n = 5])	51	20.0
Office worker (eg, clerk, typist, accountant)	25	9.8
Health worker (outside of: hospital, nurses home, nursing home [see below])	2	0.8
Rural occupation (ie, market gardener, stable hand)	2	0.8
Other (eg, labourer, storeman)	27	10.6
***Known to be in a specific hospital or other site at the time of the earthquake***		
In a hospital (public and private, employees and patients)	22	8.6
Nurses home (nurses and other employees)	10	3.9
Nursing home	17	7.0
**Total**	**255**	**100**

*Some of these people aged 65+ may still have been in paid employment (eg, self-employed). But we assumed that even if they had a stated occupation on electoral roles etc, that this was probably their pre-retirement occupation.

**Data on “occupation” for adults aged under 65 years was available for all but two cases, but for women it often included such limited terms as “married”, “spinster” and “widow” (even though some of these women may have been in the formal economy to various extents).

*** Three students died outside of school settings ie, on a shopping expedition (n = 1), and in a public toilet block (n = 2).

Overall, there were 15 buildings with three or more deaths associated with them and these accounted for 58% (126/217) of all deaths for which a location was known (Table 7). These buildings were typically constructed with brick and unreinforced masonry. An estimated 30% of deaths occurred in buildings owned by either central or local government. Overall, it appeared that deaths predominantly occurred in the business districts of Napier and Hastings and that deaths in residential homes were relatively uncommon (eg, with only 3% of deaths at home addresses).

**Table 7:** Specific establishments/locations where earthquake-related injuries occurred amongst the fatalities, including where injured outside the named premise in the street (n = 217 with data).

Name of establishment/locality	Number of deaths	Percentage	Primary building type (for specific buildings)*	Number of storeys*
***Commercial, private and other areas***				
Named commercial establishment (with 1 or 2 deaths), other than detailed below	27	12.4	—	—
Named shop (with 1 or 2 deaths per place)	23	10.6	—	—
Roach’s Department Store, Heretaunga Street, Hastings	19	8.8	Brick unreinforced masonry (URM)	2
Roman Catholic Seminary, Greenmeadows	10	4.6	Brick URM + stonework	High ceiling: equivalent to 2
Cosy Theatre Building, Hastings	10	4.6	Most likely brick URM	3
Port Ahuriri, Napier (comprising a range of buildings including wool storage sheds, owned by different companies)	8	3.7	—	—
Commercial street in Hastings (specific site on street not detailed)	7	3.2	—	—
Homes (residential)	7	3.2	Nearly all wood	Nearly all 1
Blythe’s Drapers, Emerson St, Napier	5	2.3	Most likely brick URM	3
Borthwick’s Freezing Works, Paki Paki	5	2.3	Most likely brick URM	Mix: 2 and 3
Westerman’s Store, Heretaunga Street, Hastings	4	1.8	Most likely brick URM	2
Masonic Hotel, Napier	4	1.8	Most likely brick URM	2
Commercial street in Napier (specific site on street not detailed)	4	1.8	—	—
Marcelle Toilet Rooms, Hastings (also known as McDonalds Hairdressers)	4	1.9	—	—
Grand Hotel, Heretaunga Street, Hastings	3	1.4	Brick URM	5
McGruer’s drape shop, Emerson St, Napier	3	1.4	Brick URM	2
Outside work areas	3	1.4	—	—
St John’s Anglican Cathedral	2	0.9	Brick	High ceiling: equivalent to multiple
Dr Moore’s Hospital, Marine Parade, Napier (private hospital)	1	0.5	Reinforced concrete + brick	3
Nelson Crescent Nursing Home, Napier	1	0.5	—	—
Outside area	1	0.5	—	—
**Subtotal (commercial/private/other)**	**151**	**69.5**	—	—
***Central/local government-owned facilities***				
Napier Hospital, Napier (n = 14 patients, n = 4 employees, n = 3 not detailed – either patients or visitors)	21	9.7	Most likely brick URM	Mix: 1 and 2
Park Island Old People’s Home (for the “elderly poor” and only for men after the First World War, it was built by Napier Hospital and a Charitable Aid Board)	16	7.4	Most likely brick URM	1
Napier Hospital Nurses Home Building, Napier (nurses + n = 3 clerks)	10	4.6	“Defective reinforced concrete”^8^ and soft storey	Mix: 2, 3 & 4
Technical School, Munro Street, Napier	9	4.2	Brick URM	2
Schools (particularly Greenmeadows Public School buildings + 2 other schools)	5	2.3	Greenmeadows was brick URM	—
Public library, Market Street, Hastings	3	1.4	Brick URM	2
Post Office, Hastings	2	0.9	Brick with concrete elements	2+ clock tower
**Subtotal (government facilities)**	**66**	**30.4**	—	—
**Total**	**217**	**100.0**	—	—

*Based on pre- and post-earthquake photographs in Google Images (albeit from other sources for the cathedral and the nurses home).

The predominant mechanism of death was related to building collapse (79%), followed by being struck outside (19%) eg, from chimneys or masonry falling into the street (Table 8). It is difficult to determine the role of fire, given that death from building collapse may have preceded the fire moving through a collapsed building containing people who were still alive but trapped.

**Table 8:** Estimated cause of fatal injuries (from location of death and other reports for n = 226 cases).

Estimated cause of fatal injuries	Number	Percentage
Building damage with death inside (though potentially not always associated with falling debris eg, may have been a fall from the building shaking)	140	61.9
Building damage inside or from fire (it was not possible to definitively identify deaths specifically due to the fires)	17	7.5
Death outside of a building but related to building damage (eg, falling chimneys)	41	18.1
As in the above row but with a possible role of fire	3	1.3
Death related to unspecified building damage	21	9.3
Rock fall	3	1.3
Water tank falling	1	0.4
**Total**	**226**	**100**

Napier and its suburbs experienced the most deaths (54% of the total), followed by Hastings (36%) (Table S10). Only 10% of the deaths occurred in other Hawke’s Bay towns and only 1% in rural areas.

We identified two deaths among people who went into earthquake-damaged buildings to rescue others but then were killed themselves on the day of the earthquake. But another source reports that this fatal outcome also applied to students at the Napier Technical College: “several of those students had gone back into the school to rescue trapped classmates”^4^. We found no evidence for deaths from any of the aftershocks.

## Discussion

### Main findings and interpretation

Despite New Zealand being at high risk from natural hazard events, the lack of the basic descriptive epidemiology of this major earthquake (until now) suggests a low focus on historical epidemiology for such disasters. This contrasts markedly with the epidemiology of the more slow-moving natural hazard event of the 1918/1919 influenza pandemic, for which there is detailed published New Zealand research (eg^20–23^). Given that this earthquake is probably the highest mortality *sudden natural hazard event* for people in New Zealand, it suggests that policy-makers and research funders could do more to encourage such work on, or filling gaps in, the nation’s disaster epidemiology. This conclusion also arises from the work of others^24^.

A key implication from this research is that death tolls and severe injury data for large disasters before the 21^st^ century in New Zealand, may not be particularly precise. By examining death certificates, this study identified 17 additional deaths compared with previous lists of victims (as well as discounting 14 who were on such lists). It was also able to identify five additional deaths that were indirectly related to the earthquake. These differences are not surprising, given that some people were transferred to hospitals far from the earthquake area and also some deaths occurred many months to years later (eg, in those with paraplegia). But while this study has provided a more accurate assessment than past estimates, there is still some uncertainty around the precise numbers of deaths and severe injuries attributable to this earthquake. In particular, the evidence from the Canterbury earthquake of 2011^25^ is that there may have been other cardiovascular disease-related hospital admissions and probably deaths in the year after the 1931 earthquake due to earthquake-related psychological stress.

The deaths from this earthquake occurred predominantly on the day of the earthquake (87%) and then in the subsequent week (6%) (Table 5). Of the 377 people with injury-specific details, only 4% had died by four weeks post-earthquake. However, it is unclear to the extent that this was due to low injury severity as opposed to high quality health care (albeit in the pre-antibiotic and pre-intensive care unit era).

The median age of death was 37 years (33 for females, 39.5 for males) and so using lifetable estimates for New Zealand^26^, the typical male killed lost 32 years of life span and the typical female killed lost 43 years. This equates to 9456 lost life-years for the 256 deaths in this disaster. Furthermore, this figure does not capture lost quality-of-life for those who were left disabled eg, with amputated limbs or paralysis. Nevertheless, the total impact would still have been relatively small compared to the more slowly evolving disaster of the 1918 influenza pandemic (9000 deaths)^20^ and with the country’s involvement in the First World War (18,000+ deaths)^27^.

The similarity of death rates for males and females might seem surprising, given that more women might have been located in residential settings (where death rates were low) at this period in New Zealand society eg, in childcare and other caring roles. But this factor was probably countered by the high death rates amongst women working in the retail sector (Table 6), amongst nurses (given the collapse of the hospitals and the nurses home in Napier), and the time of day when people were visiting the central city area of Napier and Hastings. The high death rate in men over 80 years is likely to be due to the collapse of the Park Island Old People’s Home where 16 men died. Similarly, the collapse of other buildings which are likely to have typically older occupants (eg, hospitals and other nursing homes) may have contributed to the higher injury and mortality rates in the older population. But it is also plausible that the higher background incidence of co-morbidities and frailty in the older population contributed to both their higher hospitalisation rates for injury and their higher mortality rates.

Perhaps the most striking feature of the mortality patterns is that there were six buildings that were associated with 10 or more deaths each (from collapse and fire) and there were 15 buildings associated with three or more deaths each (accounting for 58% of all deaths with a known location). Many of these buildings were multi-storey and involved masonry – with some of this falling into the street and killing people on the street or who ran outside during the earthquake (19% of deaths – Table 8). In contrast, the homes in residential areas which were more likely to be singled storied and be made of wood, caused relatively few deaths (3%) and there were very few deaths in rural areas.

Table 7 identified that nearly a third (30%) of the deaths occurred in buildings owned by government (central or local). This might be of particular concern given that there is a case for critical infrastructure to be subject to relatively higher building standards than that of private sector buildings. For example, hospitals are particularly important in many types of disasters and so these should ideally have been of the most robust designs. Instead, the main hospitals in Napier and Hastings both collapsed (as did a private hospital in Napier).

### Likely factors contributing to the high injury burden

The high earthquake magnitude was clearly a key factor in this disaster. Combined with this was the absence of building regulations to prevent earthquake damage or reduce injury risk. There was probably also no widespread public understanding of the risks of running immediately outside a building when an earthquake occurred. Other likely factors were as follows:

- The time of day of the earthquake (at 10.47am) probably contributed to the high mortality since people were working or shopping in the business districts of Napier and Hastings (Tables 6 and 7). Similarly, schools were in class and hospitals were probably around peak activity (Table 7).
- There was no established emergency management system in New Zealand in 1931. Nevertheless, there were many citizens with military training and experience of the mass trauma events of the First World War (including paramedics, nurses and doctors). There was also an influx of health workers from outside the region (see the Supplementary Material), and the arrival of the Royal Navy at Napier on the day of the earthquake (the naval vessel HMS *Veronica*), which meant that its crew could be deployed. It seems likely that such factors might have improved the efficiency of rescue efforts and with the operation of the field hospitals.
- Fire control capacity was limited at this time and it is possible that if fires had been extinguished earlier then more injured people could have been rescued and survived: “Ahead of the spreading fires and amidst continuing aftershocks, desperate rescuers, using crowbars, shovels, picks, and their hands, worked to reach people trapped in wrecked buildings. Some could not be rescued in time…”^4^.
- Both main hospitals in Hawke’s Bay were unusable and so field hospitals were required along with transfers to hospitals outside the region. Also at this time in history there were no antibiotics and so the case-fatality rate from infections from wounds was higher. Similarly, there were no modern intensive care units.

On the other hand, there were possibly some protective factors of relevance to this earthquake – at least compared to earthquakes in contemporary times. One was that cities at this time had fewer high rise buildings and apartments and instead the proportion of population in rural areas was higher (indeed most Māori still lived outside of cities in 1931). There were also few tourists, with only one international traveller apparently being been killed. Another factor was that it was mid-summer with fine weather, which probably improved the survival for those who were extracted from the rubble after some time, and for the injured located in tents in field hospital settings. For example, a 91 year old man was pulled alive, three days after the event^4^.

### Were some of these earthquake injuries preventable with knowledge of the day?

This question can be considered in the light of the history of documented earthquakes in New Zealand. First, it was known from the 1848 Marlborough earthquake (which was also felt in Hawke’s Bay)^28^ about the role of different building materials: “We may here remark that the damage has been sustained by those possessing brick and clay dwellings. Wooden houses in every instance have escaped”^29^. For this reason it was recommended at the time that “a public meeting of settlers should be convened, for the purpose of appointing a committee to report upon the late disastrous affliction, and to call upon the Executive to appoint an Inspector of Works so that in future buildings may be erected after some well matured and systematic plan”^29^. But this was not done, leading to the retrospective view that: “If the government of the day had taken heed of this advice it is likely that events such as the Hawke’s Bay earthquake would not have been nearly as disastrous”^29^.

Many of the stone and brick buildings in Wellington that had been destroyed in the 1848 Marlborough earthquake were nevertheless, re-erected in wood. As a result it is thought that this was a factor in the low death toll of the 1855 Wairarapa earthquake, with only between five and nine deaths, despite the large geological impact^29^. For this 1855 earthquake it was also reported that “most wooden houses, especially those of one storey, fared much better than those built of brick”^30^. A Commission after this earthquake did investigate building construction and did make recommendations for greater use of wooden framing in buildings (albeit none involving any legal requirements). The Commission also highlighted that earthquakes were a problem for the whole of the country – and not just Wellington^30^. As a result it appears that “while memories of the 1848 and 1855 earthquakes were fresh, most of the new buildings in Wellington were constructed of wood”^31^. “However, it took only 25–30 years for awareness of building safety to fade. Masonry construction gradually returned, encouraged by city council regulations for fire resistance. By the beginning of the 20^th^ century the earthquake hazard was largely discounted, and between 1913 and 1926 the *New Zealand Official Yearbook* included the comment that ‘earthquakes in New Zealand are rather a matter of scientific interest than a subject for alarm’”^31^.

In 1863 a notable earthquake actually hit Hawke’s Bay (magnitude 7.5), striking near the town of Waipukurau^32^. There were reports of numerous landslides, widespread soil liquefaction and the opening of large fissures in the ground. Houses were shaken off their piles and some chimney bricks fell through rooves^33^. However, there is no evidence that this earthquake triggered any subsequent changes in local building construction practices.

In 1929 the Murchison earthquake was felt in towns and cities throughout New Zealand^34^. A report after this earthquake also recommended that there be building standards to reduce earthquake damage, but again this advice was not substantively acted on before the 1931 Hawke’s Bay earthquake^29^.

Given the above information, it does seem likely that if the citizens of New Zealand and their government had adequately learnt from history – they may have adopted relevant building regulations before the 1931 earthquake and that this would have reduced the injury and mortality burdens. While all regulations involve trade-offs (potentially increased building costs and increased fire risk from the greater use of wood), some of the regulations may have been particularly cost-effective. For example, regulations to stabilise brick chimneys and to minimise masonry above building entrances – might have cost relatively little and yet would have saved many lives.

Furthermore, if such earthquake-related building regulations were adequately adopted and implemented throughout New Zealand, the death toll in the 2011 Canterbury earthquake would probably have been less. This is because a majority (62%) of the 185 deaths were in a single inadequately constructed building (the CTV building)^35^, and there were deaths in streets in the central city area of Christchurch (18% of all deaths)^36^, primarily from falling masonry.

The earthquake-related injury burden associated with a lack of adequate building regulations (or their implementation and enforcement), is also of relevance to modern day low-income countries in earthquake zones. These settings may wish to prioritise applying such regulations particularly to multi-storey buildings and to addressing unreinforced masonry on buildings above busy streets. For such buildings policy-makers may also wish to promote greater use of wood as a building material (with wood also providing carbon sequestration benefits, but also potentially increasing fire risk).

### Study strengths and limitations

Particular strengths of this study included it being the first to comprehensively describe the injury epidemiology of this earthquake. The study also obtained death certificates and unpublished injury related records. Nevertheless, the severe injury data are limited due to incomplete documentation, especially for cases transferred to hospitals outside the region. But even when documented, it was sometimes unclear what particular injury terms meant (eg, the extent that “shock” was from psychogenic or haemorrhagic causes). Furthermore, there was no information on the occurrence of minor injuries that did not require hospitalisations.

Even though this study benefited from considering death certificate data, at this time in history these were still not particularly detailed, were not typewritten and often were not completed as required (eg, the length of time a condition had been present was sometimes missing). They also lacked data on ethnicity. For the severe injury data we also frequently lacked occupational data and also information that linked injuries with the aftershocks that occurred in the subsequent weeks.

While the mortality data were more robust than that for hospitalised injuries, it too had limitations including missing data for the details around the location of the injury and the mechanism of injury.

## Conclusions

This earthquake remains the worst sudden mass fatality event due to a natural hazard in New Zealand’s history. This mortality burden partly related to the lack of adequate regulations for building construction to reduce earthquake risk. This was despite evidence from prior earthquakes in New Zealand (since 1848) that indicated the risks of brick and stone vs wood as construction materials. Given the experience with the Canterbury earthquake in 2011, regulations around earthquake risk continue to be of relevance for New Zealand, as well as for other countries in earthquake zones.

This study also highlights the relative lack of research into the basic epidemiology of disaster events in this country before the 21^st^ century. This suggests there is a need to upgrade funding for disaster research in this country so as to ensure that the lessons of the past are appropriately learnt.

## Supplementary information

Supplementary Material

## Data Availability

Supplemental information with additional methods and results is attached. Data sharing with other researchers or official agencies of the mortality data is available on request from the authors.
