# Supplementary material for: Descriptive Epidemiology of New Zealand’s Highest Mortality Earthquake: Hawke’s Bay in 1931
Source: Sci Rep. 2019 Mar 20;9:4914. doi: 10.1038/s41598-019-41432-6 (PMC6426931; doi:10.1038/s41598-019-41432-6)
Supplement: Supplementary file 1 — Supplementary Material [file 41598_2019_41432_MOESM1_ESM.docx]

**Supplementary Material: Additional methods details and results for the study: “Descriptive Epidemiology of New Zealand’s Highest Mortality Earthquake: Hawke’s Bay in 1931”**

Christine Clement,^1^ Shannon Abeling,^2^ Joanne Deely,^3^ Andrea Teng,^4^ George Thomson,^4^ David Johnston,^5^ Nick Wilson^4^*

^1^ Genealogist/Family Historian, Te Puke, Bay of Plenty, New Zealand

^2^ Department of Civil and Environmental Engineering, University of Auckland, Auckland, New Zealand

^3^ Canterbury District Health Board, Christchurch, New Zealand

^4^ Department of Public Health, University of Otago Wellington, New Zealand

^5^ GNS Science and Massey University, New Zealand

*Corresponding author: Professor Nick Wilson; [nick.wilson@otago.ac.nz](mailto:nick.wilson@otago.ac.nz)

**Health services provided to the injured**

As detailed in the main text, the public hospitals in Napier and Hastings were too damaged to be used. Nurses and other health workers were also killed and injured (see Tables 6 and 7). Nevertheless, there was the arrival of the Royal Navy at Napier on the day of the earthquake (the HMS *Veronica*), which meant that its crew could substantially assist with rescue efforts. The Navy also delivered 11 doctors and 17 nurses from Auckland Hospital to Napier by dawn the next day.^1^ Field hospitals were also rapidly established:

“Plans for an emergency field hospital [at the Napier racecourse] were quickly dusted off ... because it had water and was far enough inland to be out of reach of a tsunami. Four surgical teams were on site by mid-afternoon on February 3, though it was the next day before the hospital was fully set up. That did not stop emergency surgery. Doctors worked under the stark glare of car headlamps until 2am. A dressing station was also established in McLean Park.”^2^

“Doctor Duncan Stout and a large party of doctors from Wellington established a clearing station at Waipukurau.”^3^ Rail evacuation commenced on 6 February.

A police report summarised the influx of health workers into the region: “Doctors, nurses, ambulances, motor-cars, and motor-lorries arrived like magic from places between Auckland and Wellington to assist”.^4^

**Table S1: Less straight-forward and delayed deaths but which we classified as being likely to be predominantly earthquake-related (ordered by date of death)**

| **Description and interpretation** | **Date of death** | **Age-group at time of death** | **Death certificate wording** |
| --- | --- | --- | --- |
| **Person A:** No specific injury reported and she/he is reported as dying on the road. So he/she probably had an extreme stress related heart attack or stroke (ie, takotsubo cardiomyopathy). But given the lack of an autopsy it is still possible than an unreported head injury occurred. | Day of earthquake | 50-59 | Collapsed from shock shortly after earthquake - coroner considered inquest unnecessary |
| **Person B:** As the medical practitioner put “exposure to earthquake” first on the death certificate this was probably the dominant cause. Nevertheless, it is still plausible that the “frail baby difficult confinement” was more important (given that the weather conditions were warm at the time of the earthquake and there is no mention of the infant or mother being injured). | Day of earthquake | Infant | Exposure due to earthquake. Frail baby difficult confinement |
| **Person C:** The casualty record states “cerebral haemorrhage” and he/she is listed on earthquake victim lists as an earthquake fatality. Although the “11 days” was technically one day before the earthquake on 3 February, we assumed that this was a clerical mistake and that this was a cerebral haemorrhage associated with the earthquake (a head injury or a stress-induced stroke). | Mid-February 1931 | 70-79 | Cerebral haemorrhage 11 days |
| **Person D:** This person was not injured but there is reasonable evidence from the newspaper obituary that he/she experienced severe emotional shock and was subsequently hospitalised. Although the obituary notes that she/he “had not been in good health” he/she was well enough to be travelling around NZ at the time of the earthquake. This seems likely to be another case of takotsubo cardiomyopathy. | Mid-February 1931 | 50-59 | Angina pectoris, hypostatic congestion of lungs (shock and pleurisy) |
| **Person E:** This person was pulled from the ruins with injuries to hand, feet, body and face. His/her condition was described as serious (newspaper report of late February 1931). She/he developed gangrene of the feet and died in Wellington Hospital. Given the severity of these injuries it seems likely that these substantively contributed to his/her premature death. | Early March 1931 | 80+ | Heart failure (not certified) Coroner considered inquest unnecessary |
| **Person F:** This person died in New Plymouth Hospital after being transferred from Hastings. We assume that the “earthquake shock” on the death certificate was a likely cause of her/his renal failure and “uraemia”. | Early March 1931 | 70-79 | Uraemia, Senility, Earthquake shock |
| **Person G:** Given his/her relatively young age (30-39y age-group) and work location (a building known to have suffered major damage and to have caused other earthquake injuries and deaths) it is assumed that she/he suffered a head injury that resulted in a stroke (even though this person was not listed in the injury records). | Early March 1931 | 30-39 | Cerebral haemorrhage for 5 weeks |
| **Person H:** Reported to have dropped dead having been suffering from overwork since the earthquake. As a leader of a community group she/he had lost three members of that particular group. Given his/her relatively young age, it seems likely therefore that earthquake-related stress was a major factor in the cardiac condition reported on the death certificate. | Mid-March 1931 | 40-49 | Myocardial degeneration, arteriosclerosis |
| **Person I:** Reported by her/his family to have fallen off a fence and onto some object with injuries resulting. Listed in an earthquake casualty list and lists of earthquake deaths. | Mid-March 1931 | 5-9 | Intestinal Obstruction / Laparotomy Intussusception |
| **Person J:** The death certificate wording is suggestive that other causes may have predominated over the earthquake “shock”. Died in Masterton Hospital after being transferred from Napier. Nevertheless, given that “earthquake shock” is on the death certificate then this would probably have been a contributing factor. Information from a relative provided to the lead author indicated that he/she was knocked off a couch by the earthquake and sustained major injuries from this. | Late-March 1931 | 70-79 | Valvular disease, asthma and shock (earthquake) |
| **Person K:** Reported as having “slight head abrasions” in injury records. Possibly had underlying cardiac disease, nevertheless the earthquake is assumed to have been have been a contributing factor. | Early March 1931 | 20-29 | “Auricular fibrillation”, heart failure |
| **Person L:** Mentioned in Earthquake Relief Fund Cases so possibly had some injury, but it could have been due to a loss of her/his home (his/her name is not in other injury reports). While he/she probably had underlying cardiac disease, earthquake-related injury and stress is reasonably likely to have been a contributing factor in his/her cardiac condition. | Mid-April 1931 | 60-69 | Myocardial failure 1 month |
| **Person M:** The casualty reports state “shock” but also “satisfactory” at 4 weeks post-earthquake. From Napier she/he was removed to Palmerston North Hospital where he/she died. This case is somewhat uncertain but it is probably more than likely that this person had a head injury or stroke associated with the earthquake. | Early May 1931 | 60-69 | Cerebral haemorrhage (no length of time stated) |
| **Person N:** He/she was listed in the Auckland Weekly News (AWN) in late February 1931 as having a “fractured spine”, “very serious”. Burial records report her/him as a 1931 earthquake casualty. It seems more than likely that the spinal injury contributed to pyelonephritis and then premature death. | 1933 | 40-49 | Pyelonephritis 18 months, Fracture dislocation 23 months |
| **Person O:** Reported as having a “spinal injury” in casualty records. Mentioned in Earthquake Relief Fund Cases in October 1931 as “unlikely that she/he will ever leave her hospital bed”. It seems more than likely that this paralysis contributed to premature death at age 50-59. | 1935 | 50-59 | Paraplegia - 4 years and 6 months |
| **Person P:** Listed as having paralysis in casualty reports. Not listed in any previously compiled lists of earthquake-related deaths. It seems more than likely that this paralysis contributed to premature death in this 50-59 age-group. | 1936 | 50-59 | Paraplegia 6 years |
| **Person Q:** Listed in the AWN in late February 1931: “Fractured spine, very serious”. He/she was not listed in any previously compiled lists of earthquake-related deaths. It seems more than likely that this paralysis contributed to premature death at this young age. | 1944 | 30-39 | Uraemia 7 days, paraplegia due to fractured spine 13 years |

**Table S2: Deaths that we classified as probably indirectly related (ie, the earthquake is likely to have played some role but was not the predominant cause of death)**

| **Description and interpretation** | **Date of death** | **Age at time of death (y)** | **Death certificate wording** |
| --- | --- | --- | --- |
|  |  |  |  |
| **Person R:** She/he was reported to have been visiting relatives in Hastings and was injured and was taken to Auckland Hospital where she died subsequently. The obituary in a newspaper (undated) reported: “was injured at time of quake”. He/she was reported as progressing favourably but her/his condition became worse with death occurring in mid-March. The family history information suggests he/she was injured by a falling chimney. While this person’s name is not listed in official injury data it seems likely that the earthquake injury and earthquake related stress contributed to her/his stroke. Nevertheless he/she did have a preceding and long-standing cardiovascular condition and the stroke was four weeks after the earthquake. | Mid March 1931 | 50-59 | Chronic myocarditis 7 years  Cerebral haemorrhage 10 days |
| **Person S:** Her/his cardiac condition was listed first on the death certificate and it preceded the earthquake. Nevertheless, it seems likely that the stress and other problems associated with a fractured femur will have contributed to his/her cardiac condition and premature death 11 weeks after the earthquake. | Late April 1931 | 50-59 | Myocarditis 3 months, fractured femur |

**Table S3: Selected examples of deaths that we classified as not earthquake-related or which were too unclear to decide upon an earthquake role (ordered by date of death)**

| **Description and interpretation** | **Date of death** | **Age-group at time of death (y)** | **Death certificate wording** |
| --- | --- | --- | --- |
| ***Classified as not earthquake-related*** |  |  |  |
| **Person T:** This person died in Napier Hospital the day *before* the earthquake. It is understood that the body was in the hospital morgue when the building collapsed. Yet this body was buried in the Earthquake Victims Plot at Park Island Cemetery, Napier and his/her name is recorded on the Napier Earthquake Memorial at Park Island. | 2/2/1931 (day *before* the earthquake) | 10-19 | Death certificate not obtained given death preceded the earthquake |
| **Person U:** There was no reference to any injury and his/her heart condition preceded the earthquake by several days. Nevertheless, the earthquake could still have been a contributing factor (though the time of death on the day of the earthquake is not recorded). | Day of earthquake | 80+ | Cerebral haemorrhage, heart failure 3 days |
| **Person V:** She/he was transferred from Napier to Palmerston North Hospital where death occurred. Yet his/her name is on one earthquake victim list (“The Shock of ‘31”).^5^ | Mid March 1931 | 20-29 | Pulmonary tuberculosis |
| ***Classified as too unclear to attribute a role of the earthquake*** |  |  |  |
| **Person W:** He/she died in Napier Field Hospital the day after the earthquake. But given no records of injuries it is hard to know if the earthquake played any role in accelerating death from tuberculosis. | 4/2/1931 | 20-29 | Phthisis 2 years [tuberculosis] |
| **Person X:** This person had a cholecystectomy operation on the morning of the earthquake at a private hospital. The operation is likely to have been in its final stages or just completed prior to the earthquake (as per the published account of the operating doctor: D. A. Bathgate “Doctor in the sticks”. Auckland: Collins, 1972, pp162-163). She/he was transferred to the emergency hospital at Hastings Racecourse. His/her death occurred around a week later and was reported in the newspaper in the list of earthquake casualties (actually under a different name, but the newspaper subsequently published a correction). Given that pulmonary embolism is a known complication of surgery, it is unclear to us if the disruption to post-operative care from the earthquake may have contributed to the development of this complication. | Mid March 1931 | 40-49 | Pulmonary embolism,  Cholecystectomy |
| **Person Y:** Although the family is reported to have said that he/she had died as a result of earthquake injuries, there were no official records of injuries. It is unclear to us how subacute bacterial endocarditis might have been related to an earthquake injury, although perhaps she/he had pre-existing valvular heart disease from another cause and his/her heart valves were infected as a result of earthquake-related skin trauma. | 1934 | 40-49 | Subacute bacterial endocarditis |

**Table S4: Status of hospitalised cases reported by the Department of Health (with some potentially non-injured patients transferred to outside the region)***

| **Description of cases** | **Number** | **Percentage** |
| --- | --- | --- |
| Earthquake-related injury (see Table 2 for more specific detail) | 377 | 56.9 |
| Hospital transfer – no specific details | 74 | 11.2 |
| Not an injury – other diagnosis | 55 | 8.3 |
| Not stated | 148 | 22.4 |
| Unclear if diagnosis was earthquake-related | 8 | 1.2 |
| **Total** | **662** | **100** |

* Includes 4 mother child pairs – possibly with the injury or illness affecting just one of the two individuals.

**Table S5: Towns and cities that had injured earthquake victims or other patients in their hospitals with these mainly having been transported out of the Hawke’s Bay area (reported for 4 March 1931, grouped by local government region)***

| **Hospital location** | **Current Territorial Authority** | **Number of patients** | **Percentage** |
| --- | --- | --- | --- |
| Pukeora Sanatorium | Central Hawke's Bay DC | 2 | 0.3 |
| Waipukurau | Central Hawke's Bay DC | 105 | 16.2 |
| Gisborne | Gisborne DC | 1 | 0.2 |
| Hastings | Hastings DC | 2 | 0.3 |
| Levin | Horowhenua DC | 1 | 0.2 |
| Feilding | Manawatu DC | 4 | 0.6 |
| Masterton | Masterton DC | 22 | 3.4 |
| Napier (Field Hospital) | Napier CC | 49 | 7.5 |
| New Plymouth | New Plymouth DC | 2 | 0.3 |
| Palmerston North | Palmerston North CC | 173 | 26.6 |
| Taihape | Rangitikei DC | 6 | 0.9 |
| Hawera | South Taranaki DC | 22 | 3.4 |
| Dannevirke | Tararua DC | 37 | 5.7 |
| Wairoa | Wairoa DC | 14 | 2.2 |
| Wellington | Wellington CC | 140 | 21.5 |
| Wanganui | Whanganui DC | 70 | 10.8 |
| **Total** |  | **650** | **100** |

DC: District Council; CC: City Council

* This list may not be fully comprehensive as we know of a person from Auckland who was visiting Hawke’s Bay at the time of the earthquake and who was injured and then was subsequently an inpatient in Auckland Hospital.

**Table S6: Fracture sites for the 103 people who sustained one or more fractures in the earthquake***

| **Fracture site/sites** | **Number** | **Percentage** | **Compound fractures** |
| --- | --- | --- | --- |
| ***Skull, spine, chest, pelvis*** |  |  |  |
| Skull | 3 | 1.9 | 0 |
| Spine | 5 | 4.9 | 0 |
| Ribs | 11 | 10.7 | 0 |
| Pelvis | 5 | 4.9 | 1 |
| ***Upper limbs*** |  |  |  |
| “Arm” (n=8); “arms and ribs” (n=1); humerus (n=1) | 10 | 9.7 | 5 |
| Radius and ulna (n=3); radius (n=1), “forearm” (n=2) | 6 | 5.8 | 0 |
| Wrist | 1 | 1.0 | 0 |
| ***Lower limbs*** |  |  |  |
| “Leg” | 22 | 21.4 | 5 |
| Femur | 12 | 11.7 | 0 |
| Tibia (n=8); tibia + fibula (n=3) | 11 | 10.7 | 0 |
| Ankle | 3 | 2.9 | 1 |
| Foot (n=2); foot and ankle (n=1) | 3 | 2.9 | 0 |
| ***Other mixes of sites / other*** |  |  |  |
| Clavicle (n=1); “clavicle and scapula” (n=1) | 2 | 1.9 | 0 |
| “Humerus and jaw” | 1 | 1.0 | 0 |
| “Pelvis and leg” | 1 | 1.0 | 0 |
| “Leg and arm” (n=3); “leg and arms” (n=1) | 4 | 3.9 | 0 |
| “Leg and jaw” | 2 | 1.9 | 0 |
| “Skull and tibia” | 1 | 1.0 | 1 |
| Unspecified fracture | 1 | 1.0 | 0 |
| **Total** | **103** | **100** | **13** |

* This table ignores amputations some of which may have been for fractures (see Table S6); also ignored here are some of the head injuries and spinal injuries may have involved fractures (though a fracture was assumed and included here if paralysis was reported).

**Table S7: Selected demographic characteristics for the 377 earthquake-injured hospitalised cases for whom there was age/sex data**

| **Characteristic** | **Number** | **Percentage** | **Other data/analyses** |
| --- | --- | --- | --- |
| Male (based on name characteristics and out of 369 people with data) | 203 | 55.0 | The rate calculated using the urban population denominators (see Table 4) suggested that males had a non-significantly higher injury rate by 17% compared to females. |
| Female (based on name characteristics) | 166 | 45.0 |  |
| Age (mean) (for 288 individuals with data) | - | - | 41.5 years (there were no significant differences by sex)  (for age-group data, see Table S8) |
| Age (median) (for 288 individuals with data) | - | - | 42.5 years |
| Health worker occupation (nurses=7, chemist=1) out of 103 people with occupational data that could be abstracted from the electoral role | 8 | 7.8 |  |

**Table S8: Age distribution of earthquake-related hospitalisations and deaths**

| **Age-group (years)** | **Hospitalisations with age-data (see Table S7)** | | | **Deaths** | | |
| --- | --- | --- | --- | --- | --- | --- |
|  | **Number** | **Percentage** | **Rate per 1000 population** | **Number** | **Percentage** | **Rate per 1000 population** |
| 0-4 | 17 | 5.9 | 5.2 | 11 | 4.3 | 3.4 |
| 5-9 | 11 | 3.8 | 3.3 | 9 | 3.5 | 2.7 |
| 10-14 | 9 | 3.1 | 2.6 | 7 | 2.7 | 2.0 |
| 15-19 | 24 | 8.3 | 7.4 | 29 | 11.3 | 8.9 |
| 20-24 | 27 | 9.4 | 8.6 | 31 | 12.1 | 9.9 |
| 25-29 | 17 | 5.9 | 5.9 | 16 | 6.3 | 5.5 |
| 30-34 | 18 | 6.3 | 6.8 | 15 | 5.9 | 5.7 |
| 35-39 | 14 | 4.9 | 5.5 | 19 | 7.4 | 7.5 |
| 40-44 | 13 | 4.5 | 5.5 | 15 | 5.9 | 6.3 |
| 45-49 | 22 | 7.6 | 9.4 | 23 | 9.0 | 9.8 |
| 50-54 | 20 | 6.9 | 10.0 | 16 | 6.3 | 8.0 |
| 55-59 | 17 | 5.9 | 10.2 | 16 | 6.3 | 9.6 |
| 60-64 | 16 | 5.6 | 13.6 | 12 | 4.7 | 10.2 |
| 65-69 | 20 | 6.9 | 22.6 | 8 | 3.1 | 9.1 |
| 70-74 | 19 | 6.6 | 33.2 | 11 | 4.3 | 19.2 |
| 75-79 | 14 | 4.9 | 40.6 | 7 | 2.7 | 20.3 |
| 80+ | 10 | 3.5 | 40.0 | 11 | 4.3 | 44.0 |
| **Total** | **288** | **100** |  | **256** | **100** |  |

**Table S9: Locality of people with hospitalised earthquake injuries (based on available location information at the time of the earthquake or otherwise address information)**

| **Location** | **Modern day Territorial Authority** | **Number** | **Percentage** |
| --- | --- | --- | --- |
| Bay View | Napier CC | 2 | 0.6 |
| Eskdale | Napier CC | 3 | 0.9 |
| Greenmeadows | Napier CC | 12 | 3.4 |
| Meeanee | Napier CC | 4 | 1.1 |
| Napier | Napier CC | 210 | 59.2 |
| Port Ahuriri | Napier CC | 9 | 2.5 |
| Taradale | Napier CC | 6 | 1.7 |
|  | ***Subtotal*** | ***246*** | ***69.4*** |
| Hastings | Hastings DC | 72 | 20.3 |
| Havelock North | Hastings DC | 2 | 0.6 |
| Matawhi | Hastings DC | 1 | 0.3 |
| Pakowhai | Hastings DC | 1 | 0.3 |
| Tomoana | Hastings DC | 1 | 0.3 |
|  | ***Subtotal*** | ***77*** | ***21.8*** |
| Kotemaori | Wairoa DC | 4 | 1.1 |
| Mohaka | Wairoa DC | 1 | 0.3 |
| Ohuka | Wairoa DC | 1 | 0.3 |
| Putorino | Wairoa DC | 1 | 0.3 |
| Waihua | Wairoa DC | 2 | 0.6 |
| Wairoa | Wairoa DC | 5 | 1.4 |
| Willow Flat | Wairoa DC | 1 | 0.3 |
|  | ***Subtotal*** | ***15*** | ***4.3*** |
| Ongaonga | Central Hawke’s Bay DC | 1 | 0.3 |
| Otane | Central Hawke’s Bay DC | 3 | 0.9 |
| Porangahau | Central Hawke’s Bay DC | 1 | 0.3 |
| Pukehou | Central Hawke’s Bay DC | 2 | 0.6 |
| Te Aute | Central Hawke’s Bay DC | 2 | 0.6 |
| Takapau | Central Hawke's Bay DC | 2 | 0.6 |
| Waipawa | Central Hawke's Bay DC | 2 | 0.6 |
|  | ***Subtotal*** | ***13*** | ***3.9*** |
| Te Araroa | Gisborne DC* | 1 | 0.3 |
| Dannevirke | Tararua DC* | 2 | 0.6 |
| Pukapuka | Not known | 1 | 0.3 |
|  | ***Overall total*** | **355** | **100** |

* Unlikely to relate to an area of substantive earthquake damage, and so people with these home addresses may have been visiting Napier and Hasting at the time of the earthquake.

**Table S10: City, town or rural locality where earthquake-related deaths occurred (both injury related deaths and cardiovascular-related)**

| **City, town, rural locality** | **Number** | **Percentage** |
| --- | --- | --- |
| Eskdale (rural area near Napier) | 1 | 0.4 |
| Hastings City and suburbs | 91 | 35.5 |
| – Mohora (now a suburb of Hastings) | 1 | 0.4 |
| – Paki Paki (now an outlying community of Hastings) | 5 | 2.0 |
| Mohaka (rural location) | 1 | 0.4 |
| Napier City and suburbs | 137 | 53.5 |
| – Greenmeadows (now a suburb of Napier) | 14 | 5.5 |
| – Taradale (now a suburb of Napier) | 3 | 1.2 |
| Waipawa (town south of Hastings) | 1 | 0.4 |
| Wairoa (town north of Napier) | 2 | 0.8 |
| **Total** | **256** | **100** |

**References**

1 National Museum of the Royal New Zealand Navy. (1919-1939) The Navy and Disaster Relief – the 1931 Hawkes Bay Earthquake. <http://navymuseum.co.nz/1919-1939-the-navy-and-disaster-relief-the-1931-hawkes-bay-earthquake/>.

2 Wright, M. Remembering the 1931 Napier earthquake. Hawkes Bay Today (28 January 2006). <https://www.nzherald.co.nz/nz/news/article.cfm?c_id=1&objectid=10365989>.

3 Briggs, A. Report on the Earthquake at Napier, 3rd February 1931 as it Affected the Napier Hospital, and the Medical Services of the District. (24 February 1931). <http://collection.mtghawkesbay.com/search.do;jsessionid=iaoN-RvfkPliS4u1pYg--1aP?id=48578&db=object&view=detail>

4 The Police Force of the Dominion. The Police Force of the Dominion (Annual Report on). Report of Inspector Cummings, Napier District. Appendix to the Journals of the House of Representatives, 1931 Session I-II, H-16. <https://atojs.natlib.govt.nz/cgi-bin/atojs?a=d&d=AJHR1931-I-II.2.2.6.16&e=-------10--1------0-->.

5 Conly, C. The shock of '31. Auckland: Reed, 1980.
